# Interactions of the TnaC nascent peptide with rRNA in the exit tunnel enable the ribosome to respond to free tryptophan

**DOI:** 10.1093/nar/gkt923

**Published:** 2013-10-16

**Authors:** Allyson K. Martínez, Emily Gordon, Arnab Sengupta, Nitin Shirole, Dorota Klepacki, Blanca Martinez-Garriga, Lewis M. Brown, Michael J. Benedik, Charles Yanofsky, Alexander S. Mankin, Nora Vazquez-Laslop, Matthew S. Sachs, Luis R. Cruz-Vera

**Affiliations:** ^1^Department of Biology, Texas A&M University, College Station, TX 77843, USA, ^2^Department of Biological Sciences, University of Alabama in Huntsville, Huntsville, AL 35899, USA, ^3^Center for Pharmaceutical Biotechnology, University of Illinois at Chicago, Chicago, IL 60607, USA, ^4^Quantitative Proteomics Center, Department of Biological Sciences, Columbia University, New York, NY 10027, USA and ^5^Department of Biology, Stanford University, Stanford, CA 94305, USA

## Abstract

A transcriptional attenuation mechanism regulates expression of the bacterial *tnaCAB* operon. This mechanism requires ribosomal arrest induced by the regulatory nascent TnaC peptide in response to free L-tryptophan (L-Trp). In this study we demonstrate, using genetic and biochemical analyses, that in *Escherichia coli*, TnaC residue I19 and 23S rRNA nucleotide A2058 are essential for the ribosome’s ability to sense free L-Trp. We show that the mutational change A2058U in 23S rRNA reduces the concentration dependence of L-Trp-mediated *tna* operon induction, whereas the TnaC I19L change suppresses this phenotype, restoring the sensitivity of the translating A2058U mutant ribosome to free L-Trp. These findings suggest that interactions between TnaC residue I19 and 23S rRNA nucleotide A2058 contribute to the creation of a regulatory L-Trp binding site within the translating ribosome.

## INTRODUCTION

Ribosomes are cellular molecular complexes whose primary function is carrying out protein synthesis in all organisms. Prokaryote ribosomes are composed of two subunits: the small 30S subunit, which facilitates the decoding of genetic information from mRNA templates, and the large 50S subunit, which performs the polymerization of amino acids into polypeptides. Polypeptide assembly takes place in the peptidyl transferase center (PTC), which catalyzes peptide bond formation. Nascent polypeptides exit the ribosome through the peptide exit tunnel, a structure that begins at the PTC and spans the body of the large subunit.

Translation, and ultimately gene expression, can be regulated at many different levels. One of them is by direct interaction of small molecules with specific sites in the large ribosomal subunit. For instance, several antibiotics (which interfere with protein synthesis) inhibit ribosome function by binding to either the PTC or the peptide exit tunnel. Nascent peptides can also regulate the activities of the large subunit, modulating gene expression. These regulatory nascent peptides, termed ribosome arrest peptides (RAPs), induce translational arrest; the resulting arrested ribosomes control either transcription or translation of the downstream genes in the same operon (1–3).

RAPs contain specific domains, predominantly near their carboxyl termini, that are required for inducing ribosome stalling (1–5). Genetic analyses have shown that components of the ribosomal PTC and exit tunnel are required for the action of RAPs. Furthermore, structural analyses of arrested ribosomes containing RAPs show that their stalling domains form specific interactions with the PTC and the peptide exit tunnel (6). However, the exact roles of the observed interactions in inhibiting ribosome function remain obscure.

Tryptophanase is an enzyme involved in the metabolic degradation of L-tryptophan (L-Trp) (7). Tryptophanase catalyzes the breakdown of L-Trp into indole, pyruvate and ammonia. Pyruvate and ammonia are used as carbon and nitrogen sources, respectively, and indole is involved in establishing several bacterial phenotypes (8,9). In *Escherichia coli *and *Proteus vulgaris*, for example, the tryptophanase coding gene is within the tightly regulated *tnaCAB* operon. This operon contains a regulatory leader region including a small open reading frame, designated *tnaC*, which encodes the RAP TnaC, followed in the operon by two major structural genes, *tnaA,* encoding tryptophanase, and *tnaB*, encoding an L-Trp-specific transporter (10,11). Both the catabolite activator protein and free L-Trp are required to induce expression of *tnaCAB* (12,13). Although initiation of transcription of the *tnaCAB* operon is under catabolite repression control, continuation of transcription into the *tnaA* and *tnaB *structural genes is regulated by the available concentration of free L-Trp. After synthesizing the *tnaC* mRNA segment, the transcribing RNA polymerase pauses in the *tnaC–tnaA* intergenic spacer region before it can reach the *tnaA *and *tnaB* structural genes. When the cellular L-Trp levels are low, TnaC synthesis is completed, releasing TnaC and the translating ribosome at the *tnaC* stop codon. Dissociation of the ribosome allows the interaction of the Rho-termination factor with the RNA polymerase that is paused in the *tnaC–tnaA* intergenic region. This promotes premature Rho-dependent transcription termination to occur before the polymerase reaches the structural genes of the operon (13–16). Conversely, when cellular L-Trp levels are high, L-Trp is bound to the ribosome and the ribosome translating *tnaC* mRNA stalls at either the *tnaC* stop codon in *E. coli* or the *tnaC* Lys-33 codon in *P. vulgaris* (17,18). The presence of the stalled ribosome in the mRNA 5′-leader prevents the interaction of the Rho-termination factor with RNA polymerase. Therefore, transcription of the *tna* mRNA operon continues, and *tnaA* and *tnaB* are transcribed and expressed (13,16,18,19).

In bacteria that possess the *tnaCAB* operon, the specified TnaC peptides range in length from 24 to 36 amino acid residues. TnaC peptides of *E. coli* and *P. vulgaris* contain two highly conserved and one semi-conserved functional residues: a unique tryptophan residue (W12 in *E. coli *and W20 in *P. vulgaris*), a unique aspartic acid residue (D16 in *E. coli* and D24 in *P. vulgaris*) and a proline residue (P24 in *E. coli* and P32 in *P. vulgaris*) whose mutations prevent translational arrest and L-Trp-dependent *tnaCAB *operon induction (20,21). These TnaC peptides also contain a semi-conserved residue, I19 in *E. coli* and L27 in *P. vulgaris*, whose importance for TnaC function is unknown (20). It has been suggested that interactions between these TnaC residues and the ribosome promote the formation of an L-Trp binding site, at which bound L-Trp inhibits ribosome function (20). The binding of L-Trp to the TnaC-peptidyl-tRNA^Pro^-ribosome complex has been shown to block either release factor 2 (RF2)-catalyzed hydrolysis of TnaC-tRNA^Pro^ at P24 (in *E. coli*) (18) or the transfer of the TnaC peptide from TnaC-tRNA^Pro^ to Lysyl-tRNA^Lys^ at K33 (in *P. vulgaris*) (17). It has been proposed that free L-Trp binds either at or near the PTC A-site (22,23), but the exact location of the L-Trp binding site, unfortunately, remains unknown. It is also unclear how bound L-Trp inhibits ribosome function and what role(s) the TnaC peptide plays in the formation of the L-Trp binding site.

Genetic, biochemical and computational analyses have revealed possible points of interaction between the TnaC peptide and the stalled ribosome (21,22,24–26). The available data suggest that TnaC residues W12 and D16 might be involved in interactions with amino acid residues R92 and K90 of r-protein L22 and 23S rRNA nucleotides A752 and U2609 (21,24,26). Molecular dynamics simulations also suggest that TnaC amino acid residue I19 might be in contact with 23S rRNA nucleotides A2058, A2059 and U2609 (21). Cryo-EM reconstructions of the TnaC-stalled ribosome complex suggest that the stalling signal may be transmitted by a relay of TnaC–ribosome interactions from the exit tunnel to the PTC either via the TnaC peptide chain or through the ribosome, causing conformational arrangements in the PTC that impede its activity (24). Unfortunately, the cryo-EM analyses did not reveal the binding site for L-Trp (24).

In this study, we analyze the contribution of specific 23S rRNA nucleotides and TnaC residues in the ability of the ribosome to respond to varying concentrations of free L-Trp or the L-Trp analog, 1-methyl-L-Trp (1MT). Our genetic and biochemical analyses demonstrate that 23S rRNA nucleotide A2058 and TnaC residue I19 are important in promoting the sensing of L-Trp by the ribosome. Our data also indicate that Trp-tRNA^Trp^, unlike free L-Trp, does not induce either ribosome stalling or expression of the *tnaCAB* operon. We conclude from our analyses that TnaC-ribosome interactions induce the formation of a critical L-Trp binding site within the ribosome.

## MATERIALS AND METHODS

### Bacteria strains and plasmids

The *E. coli* K-12 strains and plasmids containing selected genes used in this study are listed in Supplementary Table S1. For *in vitro* assays, replacements of *tnaC* and 23S rRNA sequences were generated in the pGF2500 and pNK plasmids, respectively, using the QuikChange Lightning Site-Directed Mutagenesis Kit (Agilent Technologies). S30 cell-free extracts used in *in vitro* assays were prepared from bacterial strains with replacements of the 23S rRNA variants made as previously indicated (25). For *in vivo* assays, reporter gene mutants were obtained as follows: the *tnaC_p_tnaC-tnaA’- *region from 281 nt upstream of the *tnaC *translation start through the *BamHI* site at the *tnaA’-‘lacZ *junction were amplified from pAW137 derivatives using the primers AW217 and AW218. The PCR products were digested with *BamHI* and ligated to *BamHI*-digested pUC18. Site-directed mutagenesis to change the start codon of *tnaC *to a TGA stop codon or insert codons at *tnaC *position 25 was carried out on pUC18 derivatives using Phusion DNA polymerase and the manufacturer’s instructions (ThermoScientific). Complementary primers were designed with the desired replacements flanked by ∼10–15 nt of the wild-type sequences on each side of the change. The pUC18 plasmid derivatives that contained the desired replacements were confirmed by sequencing. *BamHI* fragments from pUC18 derivatives were ligated to *BamHI*-digested pRS552, which creates an in-frame translational fusion of *tnaA* and *lacZ*. *SalI* fragments from pRS552 derivatives containing *tnaC_p_tnaC-tnaA’-‘lacZYA* with desired *tnaC *changes were ligated to *SalI*-digested pACYC184. Finally, *SalI* fragments from pACYC184 derivatives were ligated to *XhoI*-digested pGRG36. Site-specific transposition into the *att7* locus of *E. coli* was carried out and confirmed as previously described (26,27). Bacterial strains containing mutant variants of the *tnaA’-‘lacZ* reporter genes and mutant variants of the 23S RNA gene were made as follows: pNK plasmids with desired 23S rRNA mutations were transformed into AW122 derivatives. Plating onto LB containing 100 μg/ml Amp was used to select for transformants. Transformants were picked into 2 ml LB containing 100 μg/ml Amp and incubated overnight at 37°C. In all, 10^−^^6^ dilutions of the overnight cultures were plated on LB plates containing 5% sucrose, selecting against the prrnC-sacB plasmid, and incubated overnight at 37°C (28). Colonies from LB-5% sucrose plates were replica plated onto LB plates containing either 100 μg/ml Amp or 25 μg/ml Kan. Successful replacements of prrnC-sacB with pNK derivatives were confirmed by the Amp^R^/Kan^S^ phenotype. After plasmid replacements, the 23S rRNA A2058 and A2059 mutations were verified in the strains by first amplification of 23S rRNA 1350–2902 region followed by sequencing of the resulting PCR products.

### Analysis of the effect of the A2058G mutation on protein expression in *E. coli*

The wild-type pKK3535 (29) and the mutant pKK3535A2058G plasmids were introduced into *E. coli* strain SQ171 (30). After plasmid exchange (28), the purity of the population of the mutant ribosomes in the cells carrying the pKK3535A2058G plasmid was verified by primer extension (31). For the protein analysis, cells logarithmically growing in LB medium at 37°C were harvested by centrifugation and rapidly frozen. Protein isolation and 2D-DIGE were performed as previously described (32). Proteins were extracted from the gel and identified by mass-spectrometry of the tryptic digests.

### *tnaA’-‘lacZ* reporter gene expression analysis

To analyze expression of the *tnaA’-‘lacZ* reporter gene, we performed β-galactosidase (β-gal) assays as previously described (33). β-Gal activity is reported in Miller units.

### *In vitro* accumulation of TnaC-tRNA^Pro^ and puromycin protection assays

*In vitro* reactions testing the effect of increasing concentrations of L-Trp on the accumulation of TnaC-tRNA^Pro^ were carried out as described previously (25). The reaction mixtures were performed with S30 cell-free extracts (wild-type or 23S rRNA mutants) and [^35^S]-labeled methionine. After incubation at 25°C for 5 min, 4 µg of wild-type or *tnaC* mutant mRNAs were added to the reaction mixtures. Equal aliquots of reaction mixture were added to tubes containing equal volume of L-Trp in increasing concentrations. The reaction tubes were then incubated at 37°C for 10 min, and precipitated using acetone. Dried pellets were resuspended with 25 µl of 1× loading buffer (10× Tricine loading buffer: 4% SDS, 12% glycerol, 50 mM Tris-HCl (pH 6.8), 2% 2-mercaptoethanol, 0.005% bromophenol blue) and loaded for electrophoresis on to 10% tris-tricine polyacrylamide gels.[^35^S]-labeled methionine resolved molecules were detected using autoradiography and their intensity determined with the ImageJ software. Puromycin protection assays were performed with isolated TnaC-tRNA-ribosome complexes obtained as previously indicated (25). Solutions containing [^35^S]-methionine-labeled complexes were mixed with several concentrations of L-Trp before being mixed with a puromycin solution. The final products of each reaction were resolved and analyzed as indicate earlier in text.

### Toe-printing analysis

Toe-printing assays were performed essentially as described (34), following performing cell-free transcription–translation reactions using a PURExpress system kit version (New England Biolabs.) where ribosomes were added separately and amino acid concentrations could be adjusted (Δ ribosomes, Δ amino acids). Ribosomes were isolated from the corresponding *E. coli* SQ171 described earlier in text (35). Importantly, to avoid different levels of free L-Trp background in the ribosome preparations, wild-type and mutant ribosomes were isolated in parallel using the same batches of media and buffers. The DNA templates used to direct translation contained the entire *tnaC* ORF and were PCR-amplified from wt plasmid pGF2500 or its mutant versions (described earlier in text) using forward primer T7-tnaC-2, 5′-TAATACGACTCACTATAGGGAGTTTTATAAGGAGGAAAACATATGAATATCT TACATATATGTG-3′, to add the T7 promoter sequence and an optimized translation initiation region, and reverse primer tnaC-toe-2, 5′-AGCAAACAAATAGATCACATTG-3′, which was also used as the toe-printing primer. Translation reactions were performed for 15 min at 37°C with a mixture containing every amino acid at 0.3 mM except for L-trp whose concentration was adjusted, from stock solutions in water, from zero to a range from 12.5 µM to 25 mM.

## RESULTS

**Figure 1.**
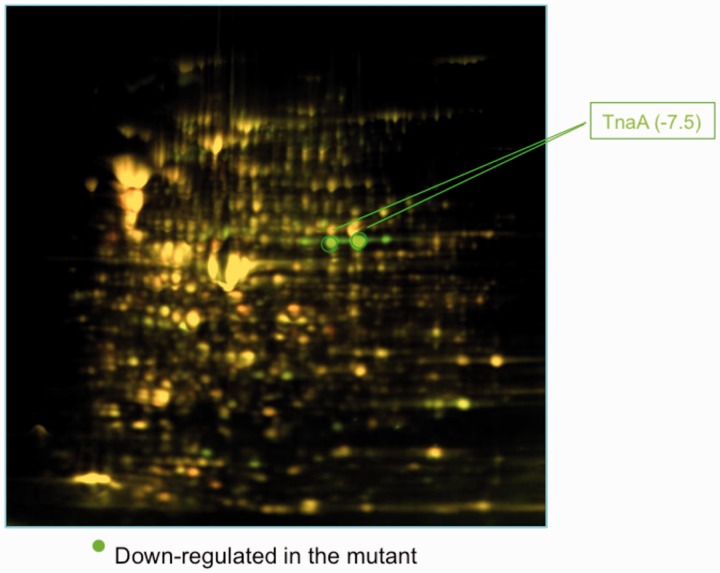
*In vivo* expression of the tryptophanase enzyme. A representative image of a 2D-DIGE experiment performed with total protein obtained from *E. coli* bacterial cells expressing wild-type 23S rRNA (protein labeled with Cy3; green) and the mutant 23S rRNA A2058G (protein labelled with Cy5; red). Green lines indicate the position of the protein bands corresponding to the tryptophanase enzyme (TnaA). The differential value in TnaA concentration between protein samples is indicated in parentheses.

The 23S rRNA nucleotides A2058 and A2059 are important for ribosome stalling induced by TnaC and L-Trp. The 23S rRNA nucleotides A2058 and A2059 are located at the surface of the exit tunnel, which makes them susceptible to interactions with nascent peptides and other ligands. These nucleotides form a hydrophobic crevice within the ribosome, which constitutes the binding site for antibiotic ligands involved in ribosome stalling regulated by the Erm leader peptides (36,37). The nucleotide A2058 also participates in translational arrest induced by the SecM RAP (36,38). In a search for other cellular proteins whose expression might depend on nascent peptide recognition in the tunnel, we used 2D-differential gel electrophoresis (2D-DIGE) to compare global expression of proteins in wild-type *E. coli* cells with those carrying the 23S rRNA A2058G mutant (‘Materials and Methods’). When cells were grown in rich (Luria–Bertani) medium, the most dramatic effect revealed by this analysis was the decreased expression of the *tnaA*-encoded tryptophanase enzyme whose steady state level in the mutant cells was reduced 7.5-fold compared with that in wild-type cells (Figure 1). This result suggested that the nucleotide A2058 plays an important role in regulating the expression of the *tnaCAB* operon.

To further investigate the role of the nucleotide A2058 and its neighboring residue A2059 in *tna* operon regulation, we prepared a library of plasmids containing mutant 23S rRNA genes with nucleotide replacements at either the A2058 or A2059 position (Supplementary Table S1). These plasmids were introduced into bacterial cells lacking chromosomal *rrn* alleles and containing a *tnaC tnaA’-‘lacZ* reporter construct (see Materials and Methods) (25,26). The engineered strains were used to analyze *in vivo* the *tnaA’-‘lacZ* reporter gene expression in response to Trp using a range of 1MT concentrations (see Materials and Methods). The 1MT is an L-Trp analog that functions as an inducer of the *tna *operon but unlike L-Trp it is not degraded or incorporated into proteins, and thus is maintained at a stable concentration within the cell (26,39). A series of graphs (β-gal activity versus 1MT concentration) were obtained to calculate the maximal induction ratio and the concentration of 1MT required to obtain 50% maximal induction. The data are summarized in Table 1. The data show that some of the mutations in the nucleotides A2058 and A2059 affected the 1MT concentration-dependence of the reporter induction. Thus, the concentration of 1MT required to obtain 50% maximal induction of the reporter in the A2058G mutant cells was 4.5 times higher than that required in wild-type cells, but maximal induction in these mutant cells reached the wild-type level at high concentrations of 1MT (Table 1). More importantly, however, both the concentration of 1MT required to obtain 50% maximal induction, and the maximal induction value are affected in the A2058U mutant cells. As seen for the A2058G mutant cells, the 1MT concentration required for 50% maximal induction in the A2058U mutant cells was four times higher than in the wild-type cells; in addition, the maximal induction was one-fourth that observed in wild-type cells (Table 1). These expression parameters were also altered in both A2059G and A2059U mutant cells, but to a lesser extent (Table 1). The previously reported U2609C change (26) had a much more profound effect on reporter expression and essentially abolished its induction (Table 1). These data indicate that changes of the nucleotides A2058 and A2059 affect the sensitivity of the reporter gene to L-Trp as inducer.

**Table 1.** Expression of the *tnaA’-‘lacZ* fusion protein in different A2058/A2059 mutant backgrounds^a^

23S rRNA	β-Gal activity^b^		1MT concentration require for 50% induction (µM)^c^	Maximal induction ratio (+1MT/−1MT)^d^
	−1MT	+1MT		
Wt	24 ± 1	1600 ± 30	7	70
A2058G	22 ± 1	1550 ± 50	32	70
A2058U	20 ± 1	360 ± 4	30	18
A2059G	42 ± 1	1950 ± 20	20	50
A2059U	20 ± 1	1040 ± 15	7	50
A2059C	25 ± 1	1200 ± 20	8	45
U2609C	25 ± 1	30 ± 1	ND	1
A2058C^e^	ND	ND	ND	ND

^a^Cultures of the following *E. coli* bacterial strains AW216 (Wt), AW680 (A2058G), AW673 (A2058U), AW676 (A2059G), AW675 (A2059U), AW677 (A2059C) and AW218 (U2609C) were grown in minimal medium supplemented with 0.2% glycerol, 0.05% acid-hydrolyzed casein, 0.01% vitamin B1 and several concentrations of 1-methyl-L-Trp (1MT).

^b^β-Galactosidase (β-Gal) assays were performed using each culture. These values are representative of three independent experiments. The maximal β-Gal values obtained for cultures grown with 1MT (+1MT) are indicated.

^c^The 1MT concentration required for 50% of induction was calculated using the data obtained from the β-Gal assays and the LMMpro non-linear regression software program version 1.06 (http://www.alfisol.com/IFS/IFS-003/LMMpro-Downloads.php).

^d^Ratio of maximal β-Gal values obtained for cultures grown with 1MT (+1MT) and those grown without 1MT (−1MT).

^e^Cells expressing A2058C 23S rRNAs are not viable.

**Figure 2.**
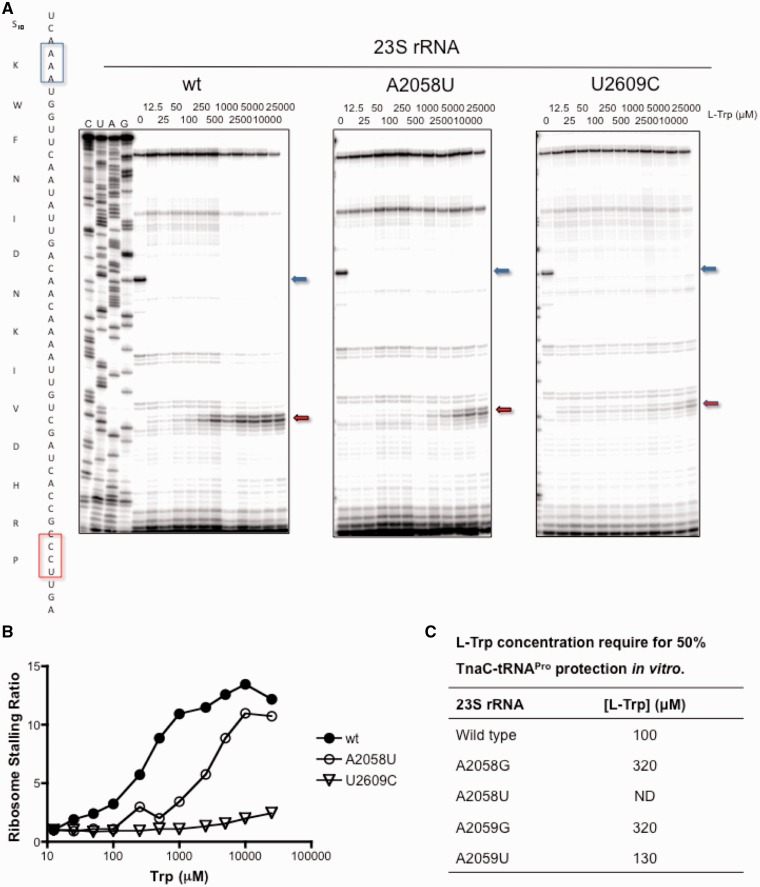
Sensitivity of mutant ribosomes for L-Trp. (**A**) Toe-printing assays performed following translation reactions in a PURE system-reconstituted with ribosomes containing the indicated 23S rRNA variants and with wild-type *tnaC* mRNAs. The TnaC peptide sequence and the *tnaC* mRNA codon sequence are shown on the left side of the figure. The positions of translational arrests caused by either the lack of Trp-tRNA^Trp^ in the system or by addition of L-Trp are indicated with boxes in the *tnaC* codon sequence, and with arrows in the right side of the autoradiograms, blue and red, respectively. (**B**) Induction plot of ribosome stalling ratio versus L-Trp (Trp) concentrations. Ratios were calculated by using the gels shown in A (which are representative of three independent experiments) and the following formula: intensity of the band corresponding to the proline codon position of each sample/intensity of the band corresponding to the proline codon position of the sample without L-Trp. (**C**) The L-Trp concentration required for 50% protection was calculated as indicted in Table 1 using the fraction of TnaC-tRNA^Pro^ [amount of TnaC-tRNA^Pro^/(amount of TnaC-tRNA^Pro ^+ amount of TnaC)] that remained in each experiment shown in Supplementary Figure S1.

We analyzed L-Trp-dependent stalling of wild-type and mutant ribosomes (A2058U or U2609C) during translation of *tnaC* mRNA performed in a cell-free transcription-translation system assembled from purified components (PURE system) (40), where the concentrations of L-Trp could be accurately adjusted (see Materials and Methods for details). Ribosome stalling at the P24 codon of *tnaC* (Figure 2A, red arrow) was monitored by toe-printing analysis over a wide range of L-Trp concentrations. In the absence of L-Trp, translation was arrested at the K11 codon because the Trp-tRNA^Trp^ level was insufficient to decode the W12 codon. Increasing the concentration of free L-Trp in the reaction mixture to 12.5 µM (lowest concentration tested) eliminated the arrest at the K11 codon, yet only negligible ribosome stalling at the P24 codon was observed because the concentration of free L-Trp was insufficient to induce formation of detectable levels of TnaC-ribosome stalled complexes. Significant ribosome stalling occurred at the P24 codon when concentrations of L-Trp were increased up to 25 mM (highest concentration tested). Remarkably, the L-Trp concentration dependence of TnaC-mediated translational arrest was shifted toward higher concentrations for the A2058U mutant compared with wild-type (Figure 2B, compare closed and open circles). In contrast, with U2609C ribosomes the stall at the P24 codon of *tnaC* was negligible, regardless of the L-Trp concentration (Figure 2B). These results indicate that the U2609C mutation affects the general mechanism of TnaC-assisted ribosome stalling, whereas the A2058U mutation decreases the affinity of the ribosome for free L-Trp.

We also tested the ability of L-Trp to inhibit PTC functions. Transfer of the nascent TnaC peptide from TnaC-tRNA^Pro^ to puromycin was monitored to determine whether L-Trp was capable of preventing peptidyl transfer in the stalled complexes composed of wild-type or mutant (A2058 or A2059) ribosomes. The concentration of L-Trp required to achieve 50% of maximal PTC inhibition was three times higher for the A2058G or A2059G ribosomes compared with wild-type ribosomes (Figure 2C), whereas no inhibition of PTC activity was observed in A2058U ribosomes when up to 1 mM L-Trp was present (the highest concentration of inducer tested in these experiments) (Supplementary Figure S1). These results verified that changes in the nucleotides A2058 and A2059 affect the concentration dependence of L-Trp-mediated inhibition of ribosome activity that underlies programmed ribosomal arrest during translation of *tnaC* mRNA.

### Features of TnaC residue I19 important for ribosome stalling induced by L-Trp

**Figure 3.**
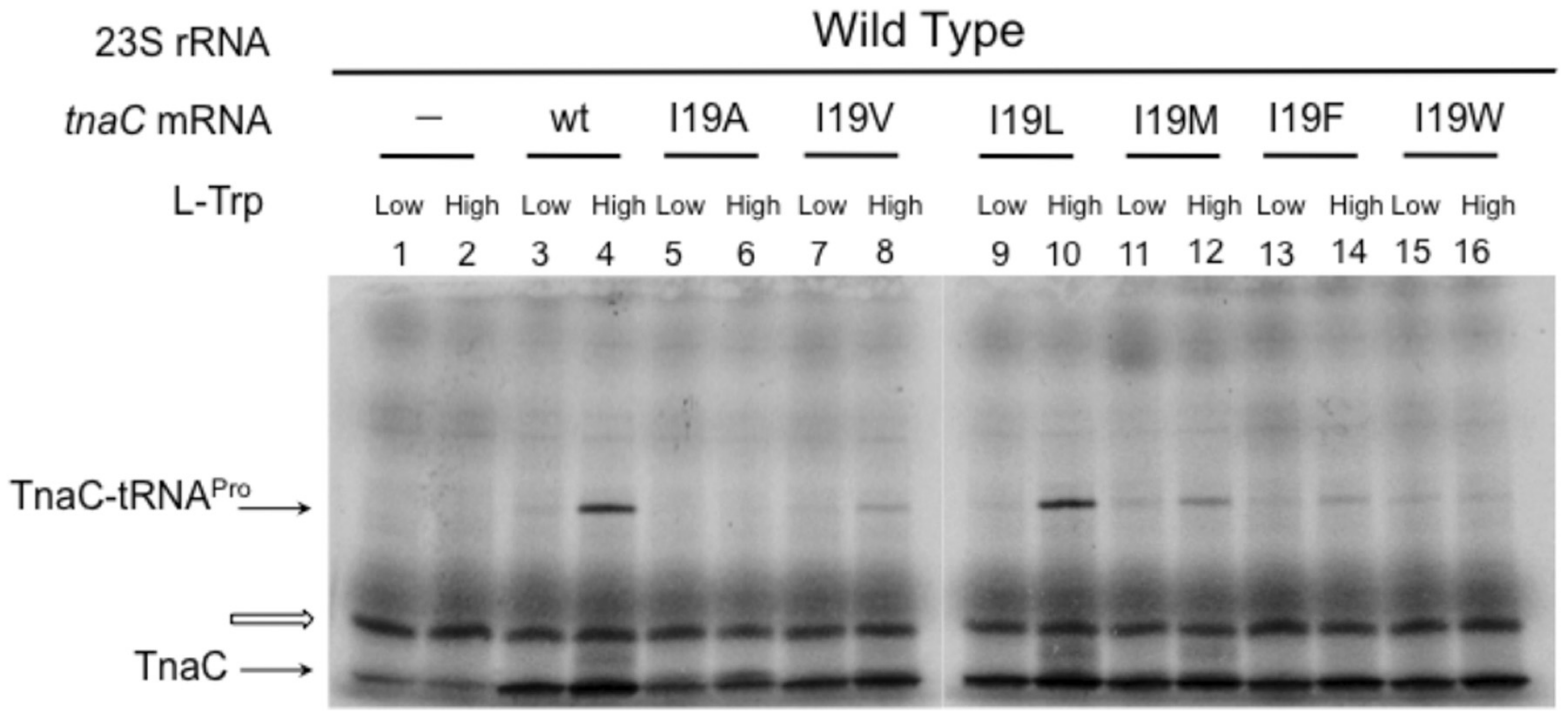
Effects of mutant TnaC peptides on the sensitivity of ribosomes for L-Trp. *In vitro* accumulation of TnaC-tRNA^Pro^ performed with wild-type cell-free extracts and the indicated *tnaC* mRNAs variants. The reactions were performed by adding (High), or not (Low), an extra 4 mM L-Trp. TnaC-tRNA^Pro^ and TnaC band positions are indicated by arrows. An unknown translated product is indicated by an open arrow.

Molecular dynamics simulations (21) that were based on the 5.8 Å cryo-EM map of the 70S-TnaC complex (24) placed TnaC residue I19 in proximity to 23S rRNA nucleotides A2058, A2059 and U2609. This TnaC residue is highly conserved among several bacterial species (20), suggesting it plays a role in Trp-dependent *tnaC* translational arrest. To test whether the TnaC residue I19 aids the ribosome in sensing the presence of the inducer, we replaced it with hydrophobic residues of variable sizes and tested these mutants *in vitro* and *in vivo*. *In vitro* formation of stalled ribosome complexes was assessed by measuring the accumulation of the TnaC-tRNA^Pro^ in *in vitro* translation systems prepared from cell-free extracts in the presence of a high (4 mM) concentration of L-Trp (Figure 3) (15). No accumulation of TnaC-tRNA^Pro^ was observed in any of the reactions containing low concentrations of L-Trp (Figure 3). With wild-type *tnaC* mRNA, as the concentration of the inducer was raised to 4 mM, a significant amount of TnaC-tRNA^Pro^ was detected, indicating that ribosomes with the wild-type TnaC nascent peptide in the exit tunnel are sensitive to L-Trp. The I19L substitution did not affect the accumulation of TnaC-tRNA^Pro^ (Figure 3, compare lane 4 with lane 10). In contrast, translation of mRNAs containing the codon changes I19V, I19M or I19F resulted in diminished accumulation of TnaC-tRNA^Pro^ compared with wild-type mRNA, whereas the I19A or I19W mutations completely abolished TnaC-tRNA^Pro^ accumulation (Figure 3, compare lane 4 with lanes 6 and 16). These results indicate that the nature of the residue at position 19 of the TnaC peptide is crucial for its ribosome arrest function in response to L-Trp.

We also examined the *in vivo* expression of *tnaC tnaA’-‘lacZ* constructs containing I19 codon replacements, at different concentrations of 1MT (Table 2). As expected, wild-type TnaC and the I19L replacement showed similar inducer dependence and required between 9–11 µM 1MT to reach 50% maximum induction. The I19A and I19W replacements, similar to the previously examined W12R substitution (26), completely abolished expression of the reporter gene. The TnaC replacements I19V and I19M not only reduced the level of maximal induction of the reporter construct but also, most remarkably, changed the sensitivity of the system to the concentration of the inducer. These TnaC mutants required 2.4 times and 6.3 times more 1MT, respectively, to obtain 50% of maximum induction compared with the wild-type construct. These results were confirmed *in vitro* by puromycin release assay (Supplementary Figure S2). Taken together, the *in vivo and in vitro* data indicate that similar to the effect of the 23 S rRNA A2058 and A2059 mutations, changes in the nature of the TnaC residue I19 alter the L-Trp concentration dependence of the ribosomal arrest during translation of *tna*C mRNA.

**Table 2.** Expression of the *tnaA’-‘lacZ* fusion protein in different *tnaC* mutant backgrounds^a^

tnaC gene	β-Gal activity		1MT concentration require for 50% induction (µM)	Maximal induction ratio (+1MT/−1MT)
	−1MT	+1MT		
Wt	120 ± 5	6800 ± 50	11	60
I19L	100 ± 4	7100 ± 60	9	70
I19M	270 ± 5	4000 ± 35	26	15
I19V	100 ± 5	1000 ± 15	69	10
I19W	796 ± 14	880 ± 5	ND	1
I19A	300 ± 2	280 ± 4	ND	1
W12R	150 ± 1	180 ± 2	ND	1

^a^Growth conditions and calculations were performed as indicated in Table 1.

The following bacterial strains AW153 (Wt), AW608 (I19L), AW607 (I19M), AW609 (I19V), AW517 (I19W), AW516 (I19A) and AW154 (W12R) were used in these experiments.

### Functional interaction between TnaC residue I19 and 23S rRNA nucleotide A2058

**Figure 4.**
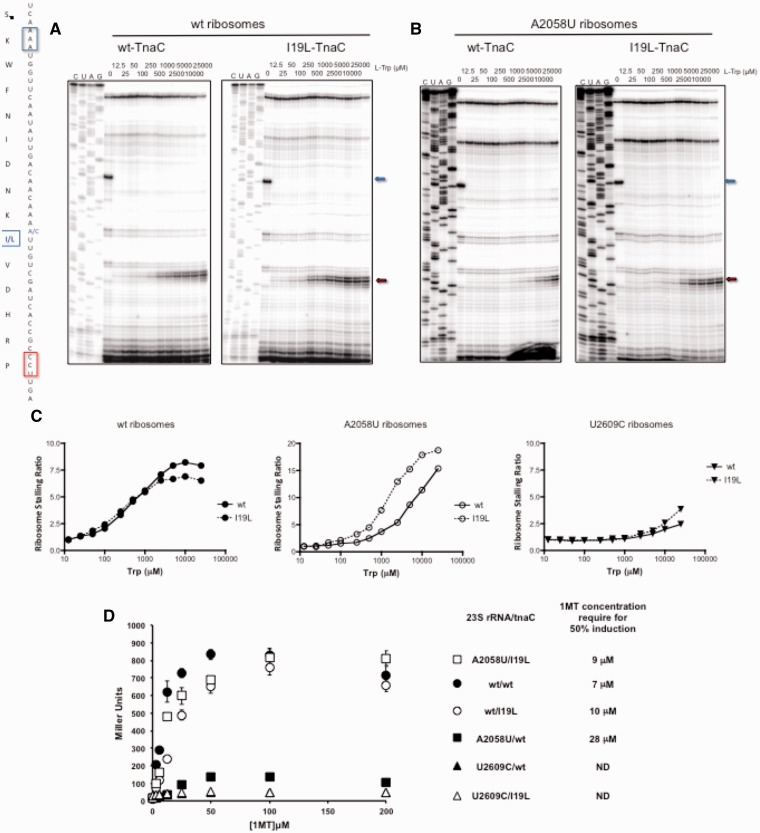
Effects of the TnaC I19L mutant peptide in the sensitivity of the A2058U mutant ribosome for L-Trp. (**A** and **B**) Toe-printing assays performed as indicated in Figure 2A. PURE system reactions were reconstituted with either wild-type ribosomes (A) or ribosomes containing 23S rRNAs with the replacement A2058U (B). *In vitro* translation reactions were performed with wild-type and I19L mutant *tnaC* mRNAs. (**C**) Induction plots were obtained as indicated in Figure 2B with the data from Figure 4A, B and Supplementary Figure S3 (these results are representative of two independent experiments). (**D**) *In vivo*-induction plot of β-gal activity (Miller Units) versus 1MT concentrations. Cultures of bacterial cells carrying a *tnaC-tnaA’-‘lacZ* reporter gene with the indicated *tnaC* alleles and expressing the indicated 23S rRNA variants were grown in minimal media under several concentration of 1MT. The 1MT concentration required for 50% induction was obtained as indicated in Table 1.

We have shown that changes of the 23S rRNA nucleotide A2058 (Table 1 and Figure 2) and of the TnaC residue I19 (Table 2 and Figure 3) affect the sensitivity of the ribosome to L-Trp. To determine whether there is an interaction between these two sensory elements, we tested whether the TnaC I19L mutation could compensate for the negative effect of the A2058U mutation on ribosome stalling. Results obtained by *in vitro* toe-printing assays showed that the I19L mutation had little effect on the Trp-concentration dependence of translational arrest induced in wild-type ribosomes (Figure 4A and C), in agreement with the results of *in vivo* and *in vitro* experiments (Figure 3). Strikingly, however, the I19L change partially restored the reduced L-Trp sensitivity of A2058U ribosomes (Figure 4B and C). This compensatory effect of the nascent peptide mutation was specific for A2058U mutant ribosomes because the same I19L mutation was unable to compensate for the negative effect on stalling of U2609C mutant ribosomes (Supplementary Figure S3).

The compensatory effect of the I19L TnaC mutation on the A2058U ribosomes was even more pronounced *in vivo*. We tested expression of the wild-type or the *tnaC*(I19L) *tnaA`-`lacZ* construct in cells containing wild-type or A2058U ribosomes (Figure 4D and Supplementary Figure S4). As we showed earlier in text (Figure 2), expression of the wild-type reporter was substantially reduced in cells containing A2058U ribosomes. However, the I19L mutational change in TnaC restored the sensitivity of A2058U ribosomes to free L-Trp. Confirming our *in vitro* results, this compensatory effect of the TnaC mutation remained specific to the A2058U mutation *in vivo* because expression of the I19L mutant reporter remained at low levels in cells with U2609C ribosomes. Altogether, these data indicate that the I19L mutational change in the TnaC nascent peptide is able to partially suppress the loss of L-Trp sensitivity of A2058U ribosomes, revealing that the ribosome and the nascent peptide cooperate in optimizing the affinity of the system for free L-Trp.

### A-site bound Trp-tRNA^Trp^ is unable to substitute for free L-Trp in translational arrest

**Figure 5.**
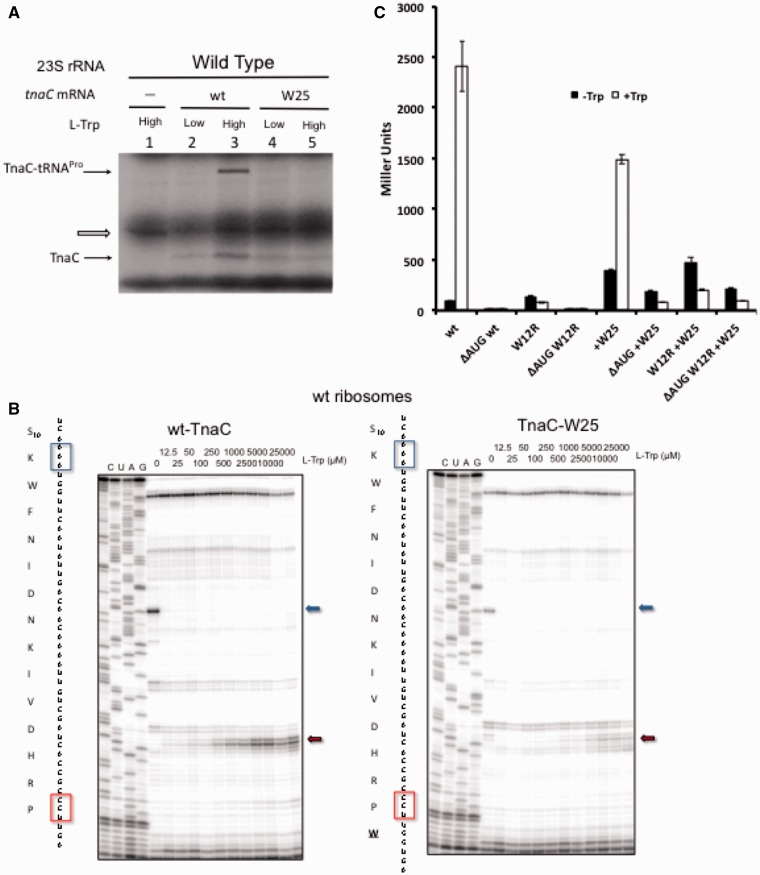
Test of tryptophanyl-tRNA^Trp^ as inducer of ribosome stalling. (**A**) *In vitro* accumulation of TnaC-tRNA^Pro^ performed with wild-type cell-free extracts with the indicated *tnaC* mRNAs variants. The reactions were performed by adding (High), or not (Low), an extra 4 mM L-Trp. TnaC-tRNA^Pro^ and TnaC band positions are indicated with arrows. An unknown translated protein is indicated by an open arrow. (**B**) Toe-printing assays performed as indicated in Figure 2A. The indicated *tnaC* mRNAs variants were translated using wild-type ribosomes. The TnaC peptide sequence and the *tnaC* codon sequence for both mRNA variants are shown on the left side of each autoradiogram. The positions of stalled ribosomes are shown with boxes in the *tnaC* codon sequence and with arrows in the right side of the autoradiograms. (**C**) *In vivo* induction plot of β-gal activity obtained from cultures of bacterial cells carrying *tnaC-tnaA’-‘lacZ* reporter genes with the indicated *tnaC* variants. The cultures were grown in the presence (+Trp) or in absence (−Trp) of 100 μg/ml L-Trp.

Although the location of the L-Trp binding site in the TnaC-stalled ribosome is unknown, it has been proposed that L-Trp may bind at or nearby the PTC A-site because Trp-tRNA^Trp^ seemed to be able to replace free L-Trp as an inducer of ribosome stalling *in vitro* (23). Our data, which show that the 23S rRNA nucleotide A2058 and the TnaC residue I19 cooperate in modulating the affinity of L-Trp for the ribosome, do not contradict this hypothesis but raise the possibility that the L-Trp binding site might be in the ribosomal peptide exit tunnel. Therefore, we revisited testing whether Trp-tRNA^Trp^ in the ribosomal A-site could substitute for the free L-Trp requirement for the translational arrest at the end of the *tna*C ORF. We performed *in vitro* translation assays by programming cell-free extracts with wild-type mRNA or mutant *tnaC* mRNA (tnaC W25) containing a Trp (UGG) codon inserted between the P24 codon and the stop codon. These experiments were carried out in cell-free extracts prepared from a bacterial strain containing the tryptophanase gene and RNAseI activity. Compared with the previous experimental set up, where coupled transcription–translation reactions were performed using cell-free extracts lacking both the tryptophanase and the RNAseI activities (15,23), our present conditions better reflect *in vivo *situations. Under our *in vitro* conditions, no accumulation of TnaC-tRNA^Pro^ was detected when *tnaC* W25 mRNA was translated irrespective of the presence or absence of exogenously added L-Trp (Figure 5A). This result argues that the binding site of free L-Trp does not coincide with the placement of the Trp moiety of the A-site bound Trp-tRNA^Trp^.

To further verify this negative result, we used *in vitro* toe-printing as an alternative independent approach (Figure 5B). If the tryptophanyl moiety of Trp-tRNA^Trp^ in the A-site can substitute for the role of free L-Trp in stalling, then even at the minimal L-Trp concentrations sufficient to bypass the W12 codon, ribosomes translating W25 mRNAs would stall at the P24 codon. However, consistent with our aforementioned results, ribosome stalling at the P24 codon of the *tnaC* W25 mRNA was nearly undetectable by toe-printing at low concentrations of L-Trp (Figure 5B). Furthermore, even at high concentrations of L-Trp, ribosomes translating the *tna*C W25 mRNA stalled less efficiently than those translating the wild-type *tna*C, confirming that addition of an extra UGG codon at the end of the *tnaC* ORF not only fails to rescue free L-Trp-independent stalling but also is even detrimental for L-Trp-induced translational arrest.

We further verified these *in vitro* results by following *in vivo* L-Trp-mediated induction of *tnaC tnaA’-‘lacZ* reporter constructs carrying the added W25 codon in the *tnaC* sequence. As seen in Figure 5C, expression of the *tnaC* W25 reporter construct retained the L-Trp dependence confirming that binding of Trp-tRNA^Trp^ to the ribosomal A-site does not substitute or abolish the role of free L-Trp as a stalling cofactor. Elimination of the *tna*C start codon (ΔAUG) or introducing the W12R replacement in the TnaC sequence eliminated the L-Trp-dependent induction of expression of both the wild-type and the W25 reporter genes. Combined, the results of these *in vitro* and *in vivo* experiments argue that the binding site of free L-Trp to the ribosome does not coincide with the position of the aminoacyl moiety of Trp-tRNA^Trp^ occupying the PTC A-site. Accordingly, the actual location of the L-Trp binding site within the ribosome remains to be determined.

## DISCUSSION

**Figure 6.**
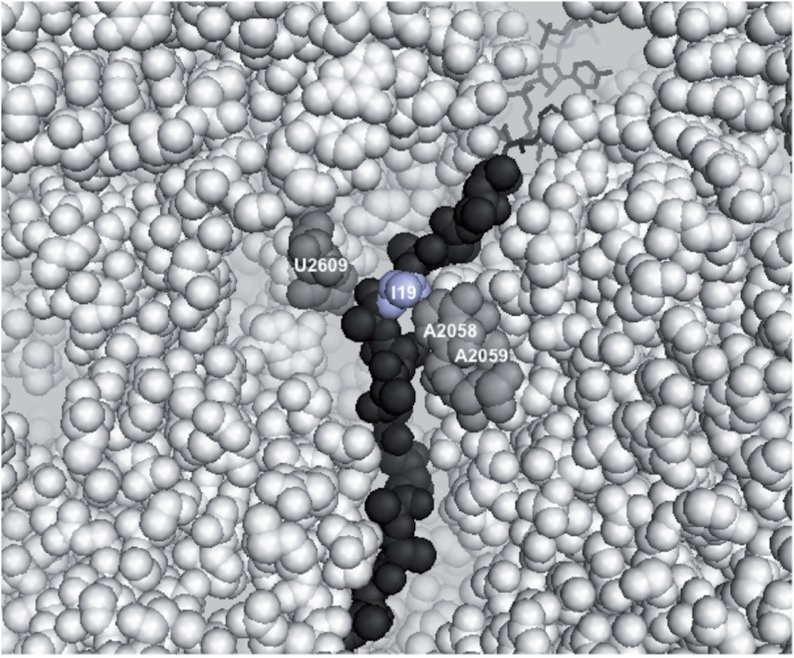
Model of the 50S ribosomal subunit bound to a TnaC-tRNA^Pro^ molecule. This model, obtained by Seidelt *et al.* (24), shows the TnaC nascent peptide (dark-gray balls) within the peptide exit tunnel. The 23S rRNA nucleotides (light-gray balls), proposed by Trabuco *et al.* (21), that could contact the TnaC residue I19 (light blue balls) are shown as well. The 3′end segment of the tRNA^Pro^ (dark-gray sticks) is shown at the PTC P-site.

In this study, we present genetic and biochemical evidence for a functional interaction between the regulatory nascent TnaC peptide and the ribosome in modulating the affinity of the ribosome for free L-Trp, the crucial cofactor in TnaC-mediated translational arrest. Mutations of either the 23S rRNA nucleotides A2058 or A2059 or the TnaC residue I19 affect the response of translational arrest to the L-Trp concentration. Importantly, however, the TnaC I19L substitution specifically suppresses the effect of the 23S rRNA A2058U mutation that reduces the affinity of the TnaC-tRNA^Pro^-ribosome complex for free L-Trp, arguing that the ribosome and the RAP cooperate in controlling properties of the L-Trp binding site. Indeed, both cryo-EM and molecular dynamics analyses suggested that the 23S rRNA residues A2058 and A2059 are in proximity to TnaC residue I19 (Figure 6) (21,24), and thus may serve as interacting partners in modulating L-Trp binding or retention in the ribosome.

Although previous data suggested that the free L-Trp binding site could coincide with the placement of the tryptophanyl moiety of Trp-tRNA^Trp^ bound in the ribosomal A-site (23), re-evaluating the previous experiments using more natural conditions and alternative approaches suggest that Trp-tRNA^Trp^ cannot substitute for free L-Trp. This, by no means, excludes the formal possibility of binding of free L-Trp in the PTC, but raises the possibility of other interpretations. One possibility is that the L-Trp binding site is formed at the site of interaction of the 23S rRNA nucleotides A2058 and A2059 with TnaC, once the I19 residue reaches these two nucleotides (Figure 6). This model, however, would presume that L-Trp binding at this site is extremely short-lived because its lifespan would be limited by the addition of P24 at the C-terminus of the nascent peptide, and peptide release on binding of the release factor RF2. An alternative, more attractive possibility is that the L-Trp binding site is formed by the residues A2058 and A2059, whereas the nascent peptide modulates the retention (dissociation rate) of L-Trp at this site. The A2058 and A2059 nucleotides form a hydrophobic crevice in the exit tunnel (37,41) where a number of ribosome-targeting antibiotics bind, including macrolides that also serve as cofactors of translational arrest mediated by leader peptides of various *erm* genes (3). Perhaps the hydrophobic planar side chain of L-Trp could be drawn into the crevice, possibly intercalating between the two adenine bases. We should note, however, that we did not observe any significant changes in reactivity of the A2058 and A2059 residues of the vacant *E. coli* ribosome to the modifying reagent dimethylsufate, even at high (5 mM) concentration of L-Trp (Klepacki and Vázquez-Laslop, unpublished results).

Alternatively, specific interactions between 23S rRNA nucleotides A2058 and A2059 and TnaC residue I19 may allosterically induce the formation of the L-Trp binding site at the PTC or another ribosomal location. Biophysical analyses suggest that residues of nascent peptides within the exit tunnel region constituted by the nucleotides A2058 and A2059 are constricted to their possible spatial conformations (42), inducing specific interactions between nascent peptides and the exit tunnel. Once specific contacts between I19 of TnaC and tunnel adenines are established, the stalling signal may be relayed to the L-Trp binding site via the nascent peptide, the ribosome or both. Relaying the conformational change from the tunnel to the PTC has been proposed for other cases of peptide-mediated translational arrest (24,34,38,43). Such a possibility would be compatible with the observation that L-Trp competes with the PTC-targeting antibiotic sparsomycin for binding to the ribosome (22). Another hydrophobic crevice formed by A2451 and C2452 in the PTC is used as a binding site by a number of antibiotic molecules (37). The properties of this crevice and its putative affinity for L-Trp could be affected by events occurring in the exit tunnel, involving the interactions between the 23S rRNA nucleotides A2058 and A2059 and the TnaC residue I19.

Regardless of the actual location of the ribosomal binding site for free L-Trp, our results clearly establish that direct interactions between the regulatory nascent TnaC peptide and the elements of the exit tunnel modulate the affinity of the ribosome for L-Trp.

## SUPPLEMENTARY DATA

Supplementary Data are available at NAR Online, including [44].

## FUNDING

National Institutes of Health USA Foundation [R01 GM47498 to M.S.S.]; Robert A. Welch Foundation [A-1310 to M.J.B.]; and National Science Foundation [MCB-1244455 to N.V.L. and A.S.M., MCB-1158271 to L.R.C.V.]. Funding for open access charge: National Science Foundation, USA [MCB-1158271].

*Conflict of interest statement*. None declared.

## Supplementary Material

Supplementary Data
