# Supplementary material for: Interactions of the TnaC nascent peptide with rRNA in the exit tunnel enable the ribosome to respond to free tryptophan
Source: Nucleic Acids Res. 2013 Oct 16;42(2):1245–56. doi: 10.1093/nar/gkt923 (PMC3902921; doi:10.1093/nar/gkt923)
Supplement: Supplementary Data [file supp_42_2_1245__index.html]

Interactions of the TnaC nascent peptide with rRNA in the exit tunnel enable the ribosome to respond to free tryptophan — Interactions of the TnaC nascent peptide with rRNA in the exit tunnel enable the ribosome to respond to free tryptophan — Supplementary Data 

# Interactions of the TnaC nascent peptide with rRNA in the exit tunnel enable the ribosome to respond to free tryptophan

## Supplementary Data

files

**Files in this Data Supplement:**

- Supplementary Data - pdf file
