# Supplementary material for: Interactions of the TnaC nascent peptide with rRNA in the exit tunnel enable the ribosome to respond to free tryptophan
Source: Nucleic Acids Res. 2013 Oct 16;42(2):1245–56. doi: 10.1093/nar/gkt923 (PMC3902921; doi:10.1093/nar/gkt923)
Supplement: Supplementary Data [file supp_gkt923_nar-02528-r-2013-File008.pdf]

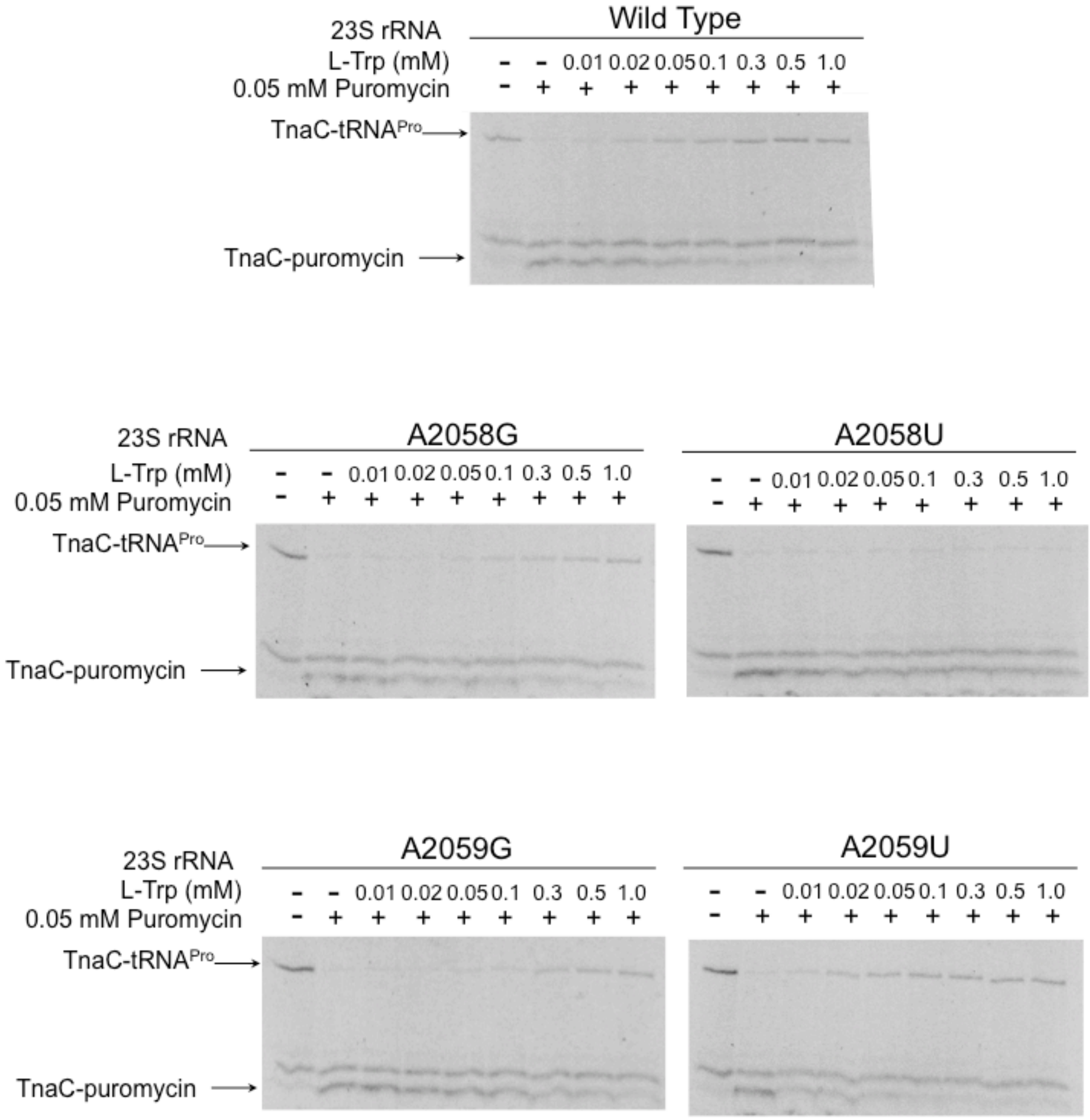

Figure S1. L-Trp-protection assays performed with stalled ribosome complexes containing wild type *tnaC* mRNA and the indicated 23S rRNAs. Puromycin-cleavage of TnaC-tRNA^{Pro} molecules was challenged with the indicated L-Trp concentrations. TnaC-tRNA^{Pro} and TnaC band positions are indicated by arrows.

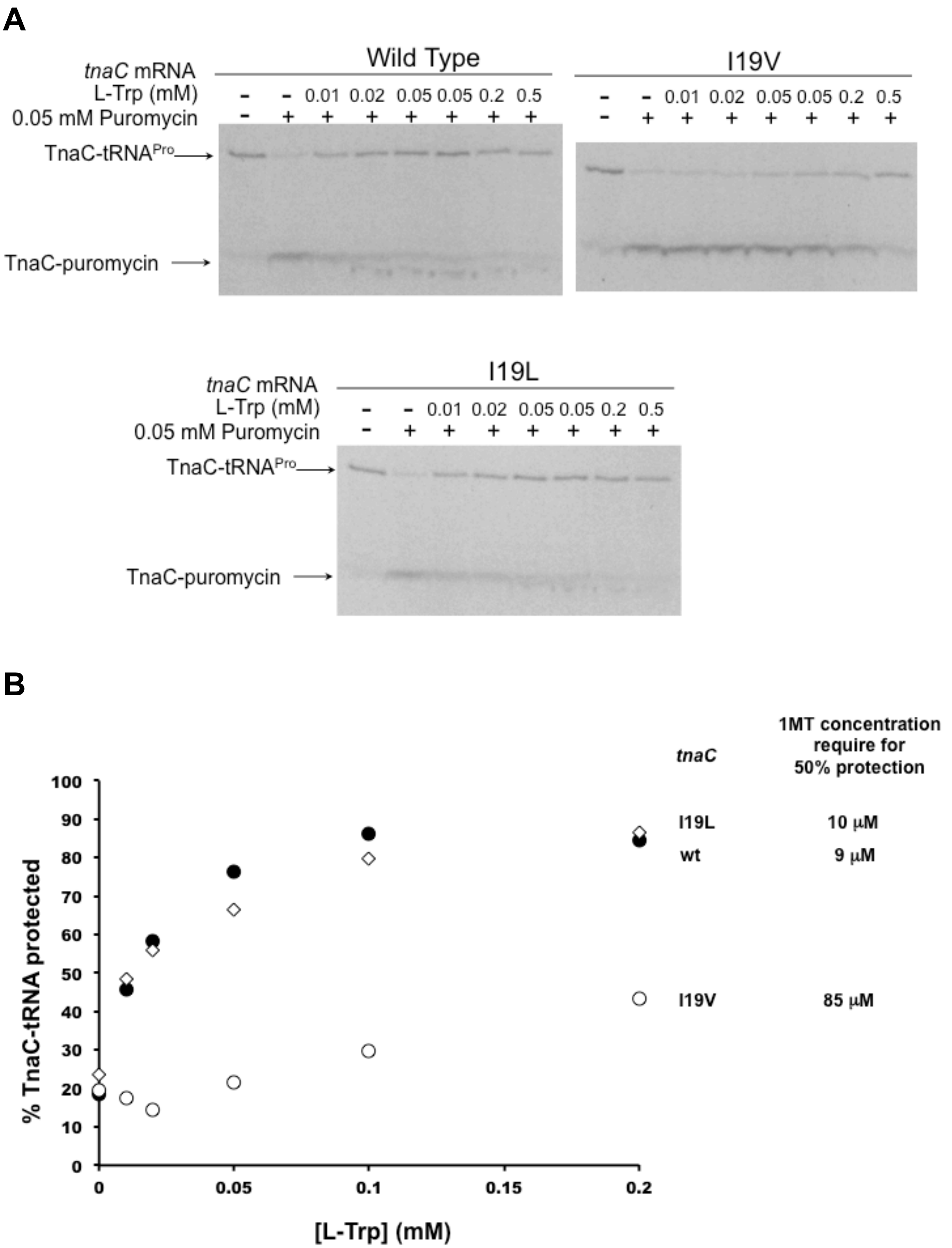
A
Wild Type
I19V
tnaC mRNA
L-Trp (mM)
- - 0.01 0.02 0.05 0.05 0.2 0.5
0.05 mM Puromycin
- + + + + + + +
TnaC-tRNA^Pro
TnaC-puromycin
I19L
B
% TnaC-tRNA protected
[L-Trp] (mM)
tnaC
1MT concentration require for 50% protection
I19L 10 μM
wt 9 μM
I19V 85 μM
100
90
80
70
60
50
40
30
20
10
0
0
0.05
0.1
0.15
0.2

Figure S2. A) L-Trp protection analyses using isolated stalled ribosomes. Stalled ribosome complexes were isolated from *in vitro* translation reactions performed with RF2-depleted cell free extracts and the indicated biotinylated *tnaC* mRNAs. The isolated complexes were mixed, or not (-), with the indicated concentrations of L-Trp prior to the addition (+) of 0.05 mM puromycin. Products of the reactions were resolved by electrophoresis in 10% tris-tricine polyacrilamide gels. TnaC-tRNA^{Pro} and TnaC-puromycin molecule positions are shown by arrows. B) Plot of the percent (%) of TnaC-tRNA^{Pro} protected vs L-tryptophan concentrations. The values of % of TnaC-tRNA^{Pro} protected were obtained from the figures on A) using the following formula: The percent (%) of TnaC-tRNA^{Pro} that remained in each experiment was calculated using the following formula: % of TnaC-tRNA^{Pro} = [amount of remained TnaC-tRNA^{Pro}/ (amount of remained TnaC-tRNA^{Pro} + amount of TnaC)].

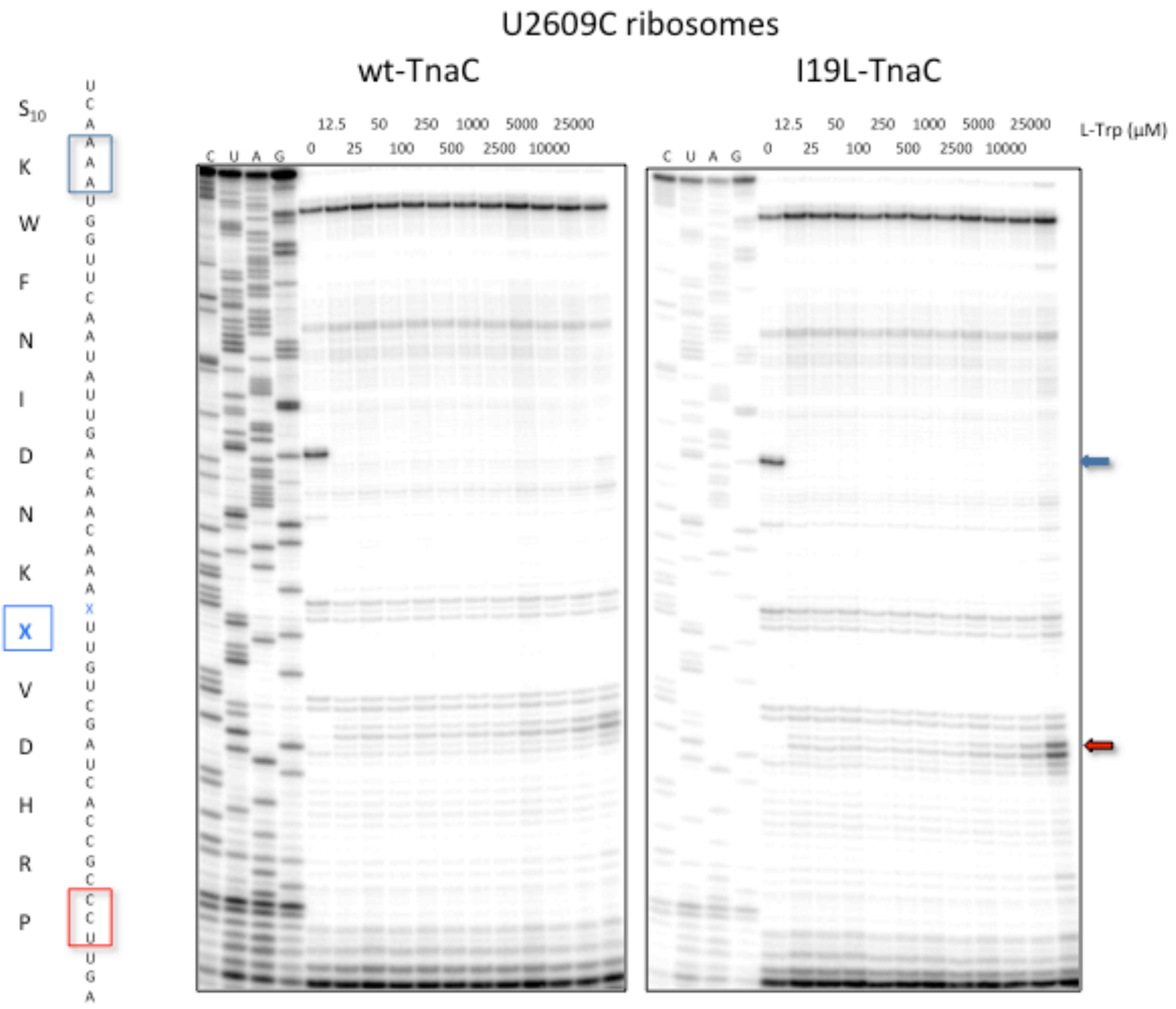

Figure S3. Toe-printing analyses performed as indicated in Figure 2A. Reactions were performed with mutant U2609C ribosomes and the indicated *tnaC* mRNAs.

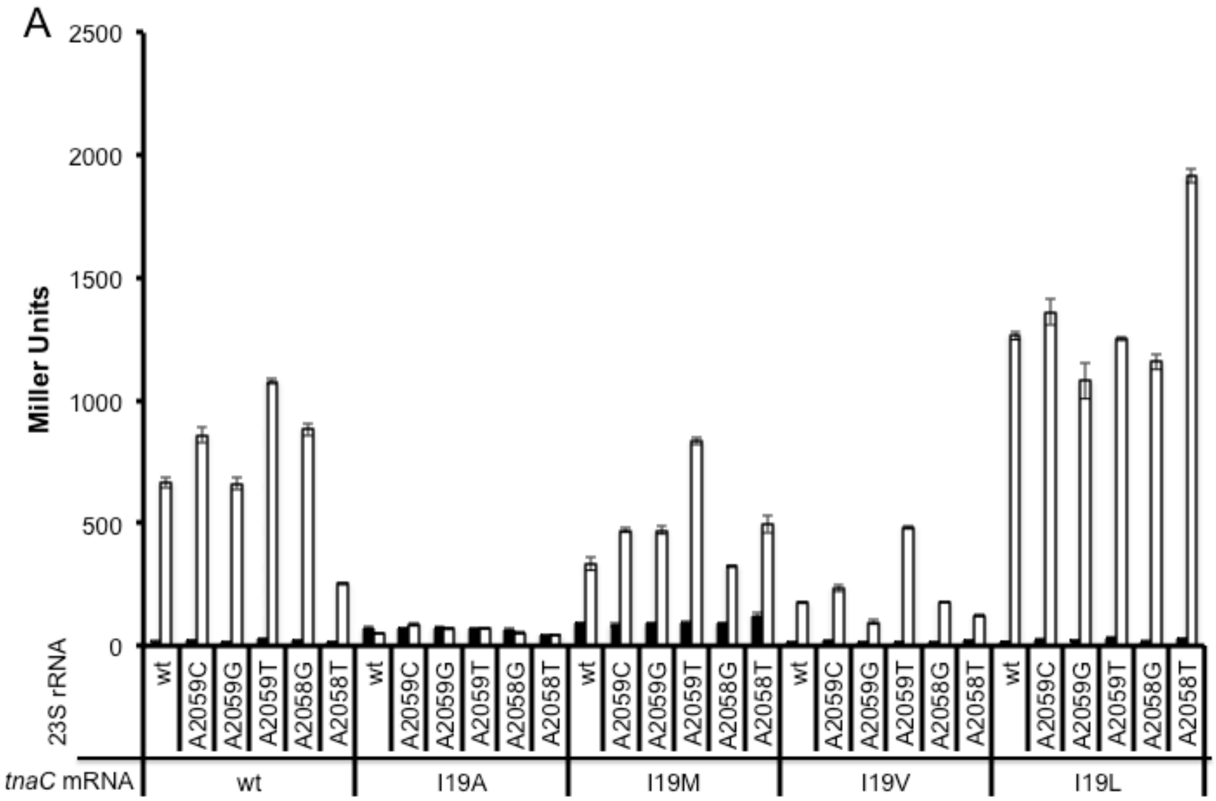

Figure S4. A) Bacterial cells expressing the indicated 23S rRNA's and *tnaC* alleles were used to analyze expression of β-galactosidase from a strain expressing a *tnaC-tnaA'-'lacZ* protein fusion. The tested cultures were grown in minimal medium containing 0.2% glycerol, 0.05% acid-hydrolyzed casein, 0.01 % vitamin B1, in the presence several concentrations of 1MT.

Table S1. *E. coli* bacterial plasmids and strains used in this work.

Plasmid	Description	Source
ptRNA67	tRNA encoding plasmid	28
prrnC-sacB	Wild-type *rrnC* operon; Km^r, and a *sacB* gene, derived from pCS101	28
pNK	Wild-type *rrnB* operon; Amp^r, derived from ColE1	38
pKK3535	Wild-type *rrnB* operon; Amp^r, derived from ColE1	29
pKK3535 2058G	Derived from pKK3535 - has an A-to-G replacement at position 2058 in 23S rRNA	This study
pNKA2058G	Derived from pNK - has a A-to-G replacement at position 2058 of the 23S rRNA	This study
pNKA2058T	Derived from pNK - has an A-to-T replacement at position 2058 in 23S rRNA	This study
pNKA2059C	Derived from pNK has a A-to-C replacement at position 2059 in 23S rRNA	This study
pNKA2059G	Derived from pNK - has an A-to-G replacement at position 2059 in 23S rRNA	This study
pNKA2059T	Derived from pNK has a A-to-T	This study

	replacement at position 2059 in 23S rRNA	
pKKU2609C	Derived from pNK has a T-to-C replacement at posiiton 2609 in 23S rRNA	44
pAW137	Has *tna_ptnaC(ΔN2-H22)* with the BsaI-XhoI-BsaI linker-*tnaA'-'lacZYA* cloning reporter gene derived from pACYC184	26

Strain	Description	Source
SQ171	MG1655 λ⁻ *rph-1 Δ(rrsH-aspU)794(::FRT) Δ(rrfG-rrsG)791(::FRT) Δ(rrfF-rrsD)793(::FRT) Δ(rrsC-trpT)795(::FRT) Δ(rrsA-rrfA)792(::FRT) Δ(rrsB-rrfB)790(::FRT) Δ(rrsE-rrfE)789(::FRT)*	30
AW122	Derived from SQ351 (p*rrnC-sacB*, ptRNA67)	26
AW153	MG1655 Δ(*lacZYA*) *att7::tna$_p$tnaC*(*tnaA*'-'*lacZYA*)	26
AW154	MG1655 Δ(*lacZYA*) *att7::tna$_p$tnaC(W12R)*(*tnaA*'-'*lacZYA*)	26
AW516	MG1655 Δ(*lacZYA*) *att7::tna$_p$tnaC*(*I19A*)(*tnaA*'-'*lacZYA*)	This study

AW517	MG1655 Δ(*lacZYA*) *att7::tna*$_p$*tnaC*(*I19W*)(*tnaA*'-'*lacZYA*)	This study
AW607	MG1655 Δ(*lacZYA*) *att7::tna*$_p$*tnaC*(*I19M*)(*tnaA*'-'*lacZYA*)	This study
AW608	MG1655 Δ(*lacZYA*) *att7::tna*$_p$*tnaC*(*I19L*)(*tnaA*'-'*lacZYA*)	This study
AW609	MG1655 Δ(*lacZYA*) *att7::tna*$_p$*tnaC*(I19V)(*tnaA*'-'*lacZYA*)	This study
AW643	MG1655 Δ(*lacZYA*) *att7::tna*$_p$*tnaC(ΔAUG)*(*tnaA*'-'*lacZYA*)	This study
AW747	MG1655 Δ(*lacZYA*) *att7::tna*$_p$*tnaC(W12R)(ΔAUG)*(*tnaA*'-'*lacZYA*)	This study
AW221	MG1655 Δ7 *rrn* Δ(*lacZYA*) *att7::tna*$_p$*tnaC(W12R)*(*tnaA*'-'*lacZYA*) (pNK, ptRNA67)	26
AW216	MG1655 Δ7 *rrn* Δ(*lacZYA*) *att7::tna*$_p$*tnaC*(*tnaA*'-'*lacZYA*) (pNK, ptRNA67)	26
AW677	MG1655 Δ7 *rrn* Δ(*lacZYA*) *att7::tna*$_p$*tnaC*(*tnaA*'-'*lacZYA*) (pNKA2059C, ptRNA67)	This study
AW676	MG1655 Δ7 *rrn* Δ(*lacZYA*) *att7::tna*$_p$*tnaC*(*tnaA*'-'*lacZYA*) (pNKA2059G, ptRNA67)	This study

AW675	MG1655 Δ7 *rrn* Δ(*lacZYA*) *att7::tna*$_p$*tnaC*(*tnaA*'-'*lacZYA*) (pNKA2059T, ptRNA67)	This study
AW680	MG1655 Δ7 *rrn* Δ(*lacZYA*) *att7::tna*$_p$*tnaC*(*tnaA*'-'*lacZYA*) (pNKA2058G, ptRNA67)	This study
AW673	MG1655 Δ7 *rrn* Δ(*lacZYA*) *att7::tna*$_p$*tnaC*(*tnaA*'-'*lacZYA*) (pNKA2058T, ptRNA67)	This study
AW701	MG1655 Δ7 *rrn* Δ(*lacZYA*) *att7::tna*$_p$*tnaC*(*I19A*)(*tnaA*'-'*lacZYA*) (pNK, ptRNA67)	This study
AW684	MG1655 Δ7 *rrn* Δ(*lacZYA*) *att7::tna*$_p$*tnaC(I19A)*(*tnaA*'-'*lacZYA*) (pNKA2059C, ptRNA67)	This study
AW681	MG1655 Δ7 *rrn* Δ(*lacZYA*) *att7::tna*$_p$*tnaC(I19A)*(*tnaA*'-'*lacZYA*) (pNKA2059G, ptRNA67)	This study
AW683	MG1655 Δ7 *rrn* Δ(*lacZYA*) *att7::tna*$_p$*tnaC(I19A)*(*tnaA*'-'*lacZYA*) (pNKA2059T, ptRNA67)	This study
AW682	MG1655 Δ7 *rrn* Δ(*lacZYA*) *att7::tna*$_p$*tnaC(I19A)*(*tnaA*'-'*lacZYA*) (pNKA2058G, ptRNA67)	This study
AW685	MG1655 Δ7 *rrn* Δ(*lacZYA*) *att7::tna*$_p$*tnaC(I19A)*(*tnaA*'-'*lacZYA*)	This study

	(pNKA2058T, ptRNA67)	
AW691	MG1655 Δ7 *rrn* Δ(*lacZYA*) *att7::tna$_p$tnaC*(*I19M*)(*tnaA*'-'*lacZYA*) (pNK, ptRNA67)	This study
AW692	MG1655 Δ7 *rrn* Δ(*lacZYA*) *att7::tna$_p$tnaC(I19M)*(*tnaA*'-'*lacZYA*) (pNKA2059C, ptRNA67)	This study
AW694	MG1655 Δ7 *rrn* Δ(*lacZYA*) *att7::tna$_p$tnaC(I19M)*(*tnaA*'-'*lacZYA*) (pNKA2059G, ptRNA67)	This study
AW693	MG1655 Δ7 *rrn* Δ(*lacZYA*) *att7::tna$_p$tnaC(I19M)*(*tnaA*'-'*lacZYA*) (pNKA2059T, ptRNA67)	This study
AW700	MG1655 Δ7 *rrn* Δ(*lacZYA*) *att7::tna$_p$tnaC(I19M)*(*tnaA*'-'*lacZYA*) (pNKA2058G, ptRNA67)	This study
AW695	MG1655 Δ7 *rrn* Δ(*lacZYA*) *att7::tna$_p$tnaC(I19M)*(*tnaA*'-'*lacZYA*) (pNKA2058T, ptRNA67)	This study
AW704	MG1655 Δ7 *rrn* Δ(*lacZYA*) *att7::tna$_p$tnaC*(*I19V*)(*tnaA*'-'*lacZYA*) (pNK, ptRNA67)	This study
AW705	MG1655 Δ7 *rrn* Δ(*lacZYA*) *att7::tna$_p$tnaC(I19V)*(*tnaA*'-'*lacZYA*) (pNKA2059C, ptRNA67)	This study
AW706	MG1655 Δ7 *rrn* Δ(*lacZYA*)	This study

	att7::tna_p*tnaC(I19V)*(*tnaA*’-‘*lacZYA*)<br>(pNKA2059G, ptRNA67)	
AW707	MG1655 Δ7 *rrn* Δ(*lacZYA*)<br>*att7::*tna_p*tnaC(I19V)*(*tnaA*’-‘*lacZYA*)<br>(pNKA2059T, ptRNA67)	This study
AW708	MG1655 Δ7 *rrn* Δ(*lacZYA*)<br>*att7::*tna_p*tnaC(I19V)*(*tnaA*’-‘*lacZYA*)<br>(pNKA2058G, ptRNA67)	This study
AW709	MG1655 Δ7 *rrn* Δ(*lacZYA*)<br>*att7::*tna_p*tnaC(I19V)*(*tnaA*’-‘*lacZYA*)<br>(pNKA2058T, ptRNA67)	This study
AW711	MG1655 Δ7 *rrn* Δ(*lacZYA*)<br>*att7::*tna_p*tnaC*(*I19L*)(*tnaA*’-‘*lacZYA*) (pNK,<br>ptRNA67)	This study
AW712	MG1655 Δ7 *rrn* Δ(*lacZYA*)<br>*att7::*tna_p*tnaC(I19L)*(*tnaA*’-‘*lacZYA*)<br>(pNKA2059C, ptRNA67)	This study
AW713	MG1655 Δ7 *rrn* Δ(*lacZYA*)<br>*att7::*tna_p*tnaC(I19L)*(*tnaA*’-‘*lacZYA*)<br>(pNKA2059G, ptRNA67)	This study
AW714	MG1655 Δ7 *rrn* Δ(*lacZYA*)<br>*att7::*tna_p*tnaC(I19L)*(*tnaA*’-‘*lacZYA*)<br>(pNKA2059T, ptRNA67)	This study
AW715	MG1655 Δ7 *rrn* Δ(*lacZYA*)<br>*att7::*tna_p*tnaC(I19L)*(*tnaA*’-‘*lacZYA*)<br>(pNKA2058G, ptRNA67)	This study

AW724	MG1655 Δ7 *rrn* Δ(*lacZYA*) *att7::tna$_p$tnaC(I19L)*(*tnaA*'-'*lacZYA*) (pNKA2058T, ptRNA67)	This study
AW218	MG1655 Δ7 *rrn* Δ(*lacZYA*) *att7::tna$_p$tnaC*(*tnaA*'-'*lacZYA*) (pNH2609, ptRNA67)	26
AW814	MG1655 Δ7 *rrn* Δ(*lacZYA*) *att7::tna$_p$tnaC(I19L)*(*tnaA*'-'*lacZYA*) (pNH2609, ptRNA67)	This study
AW671	MG1655 Δ(*lacZYA*) *att7::tna$_p$tnaC(+W25)*(*tnaA*'-'*lacZYA*)	This study
AW672	MG1655 Δ(*lacZYA*) *att7::tna$_p$tnaC(+W25)(ΔAUG)*(*tnaA*'-'*lacZYA*)	This study
AW772	MG1655 Δ(*lacZYA*) *att7::tna$_p$tnaC(W12R)(+W25)*(*tnaA*'-'*lacZYA*)	This study
AW752	MG1655 Δ(*lacZYA*) *att7::tna$_p$tnaC(W12R)(+W25)(ΔAUG)*(*tnaA*'-'*lacZYA*)	This study
AW778	MG1655 Δ(*lacZYA*) *att7::tna$_p$tnaC(+I25-AUU)*(*tnaA*'-'*lacZYA*)	This study
AW755	MG1655 Δ(*lacZYA*) *att7::tna$_p$tnaC(+I25-AUU)(ΔAUG)*(*tnaA*'-'*lacZYA*)	This study
AW750	MG1655 Δ(*lacZYA*) *att7::tna$_p$tnaC(+I25-AUA)*(*tnaA*'-'*lacZYA*)	This study

AW801	MG1655 Δ(*lacZYA*) *att7::tna$_p$tnaC(+I25-AUA)(ΔAUG)*(*tnaA*'-'*lacZYA*)	This study
AW826	MG1655 Δ(*lacZYA*) *att7::tna$_p$tnaC(+stop25)*(*tnaA*'-'*lacZYA*)	This study

Supplementary References

44. Garza-Ramos, G., Xiong, L., Zhong, P. and Mankin AS. (2001). Binding site of macrolide antibiotics on the ribosome: new resistance mutation identifies a specific interaction of ketolides with rRNA. *J. Bacteriol,* **183**, 6898-6907.
